# Poly(silyl ether)s as Degradable and Sustainable Materials: Synthesis and Applications

**DOI:** 10.3390/molecules29071498

**Published:** 2024-03-27

**Authors:** Vladimir Zotov, Srikanth Vijjamarri, Seyed-Danial Mousavi, Guodong Du

**Affiliations:** Department of Chemistry, University of North Dakota, 151 Cornell Street Stop 9024, Grand Forks, ND 58202, USA; vladimir.zotov@und.edu (V.Z.); srikanth.bns07@gmail.com (S.V.);

**Keywords:** Poly(silyl ether), degradable polymers, sustainable materials, polymers, hydrolytic degradability, catalytic hydrosilylation

## Abstract

Polymer research is currently focused on sustainable and degradable polymers which are cheap, easy to synthesize, and environmentally friendly. Silicon-based polymers are thermally stable and can be utilized in various applications, such as columns and coatings. Poly(silyl ether)s (PSEs) are an interesting class of silicon-based polymers that are easily hydrolyzed in either acidic or basic conditions due to the presence of the silyl ether Si-O-C bond. Synthetically, these polymers can be formed in several different ways, and the most effective and environmentally friendly synthesis is dehydrogenative cross coupling, where the byproduct is H_2_ gas. These polymers have a lot of promise in the polymeric materials field due to their sustainability, thermal stability, hydrolytic degradability, and ease of synthesis, with nontoxic byproducts. In this review, we will summarize the synthetic approaches for the PSEs in the recent literature, followed by the properties and applications of these materials. A conclusion and perspective will be provided at the end.

## 1. Introduction

**Scheme 1:**
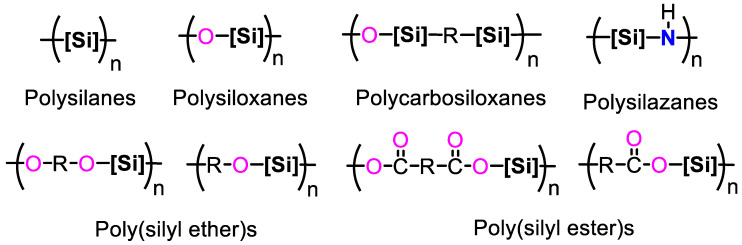
Main types of silicon-containing polymers (where **[Si]** = SiRR’ or SiR_2_-X-SiR_2_).

Silicon-containing polymeric materials have received much attention in organic and material chemistry due to their various industrial and academic applications [1,2,3,4,5,6,7]. Based on the silicon linkages of the main chains, these materials can be classified into several major types (Scheme 1). Polysilanes with –(Si)_n_– repeating units are promising in electronic devices because of their conductivity [8]. Polysilazanes are a type of silicon-containing polymers with silicon–nitrogen bonds as backbones. These polymers are known for their high temperature and oxidative stability, high hardness, and high resistance to many chemicals. Because of these properties, they have been employed as materials in silicon-based ceramic products, such as fibers, coatings, and fiber-reinforced ceramic–matrix composites. Poly(siloxane) backbones are formed by alternating silicon and oxygen atoms (–(Si-O)_n_–). Because of their inherent properties, particularly their low-temperature flexibility and high-temperature stability, poly(siloxane)s are the most widely used silicon-containing polymers, as elastomers, plastics, and in other industrial applications [9,10,11,12,13,14,15,16]. In addition, poly(siloxane)s are important precursors of silicon ceramics [17,18], such as silicon oxycarbides (SiOCs), which can be applied in coatings and as electrode materials in lithium batteries [19,20,21,22,23,24]. Polycarbosiloxanes with –(Si-R-Si-O)_n_– linkages allow various organic components to be introduced in their main chains, greatly expanding the structural diversity of the polymers. While poly(siloxane)s and polycarbosiloxanes are known and used for their stability, poly(silyl ether)s (PSEs) and poly(silyl ester)s [25,26] are hydrolytically degradable silicon polymers because of the presence of labile Si-O-C and CO-O-Si linkages in their polymer backbones. In particular, PSEs are high-performance materials and display excellent low-temperature flexibilities along with greater thermal stabilities. Importantly, the degradation behavior and the thermomechanical properties of PSEs can be adjusted by changing substituent groups on silicon and/or carbon backbones [27,28,29] and by copolymerization with other segments. These combined features have led to their broad implementation as elastomers, gas-permeable membranes, biocompatible coatings, and materials in drug delivery processes.

Both silicon and oxygen are among the most abundant elements on Earth and are practically inexhaustible. Carbon has been conventionally derived from petroleum resources and can be more readily sourced from biomass. Furthermore, biomass is rich in carbon and oxygen, which typically requires energy-intensive deoxygenation steps in the biomass conversion processes. PSEs provide the possibility of incorporating both carbon and oxygen directly into end products. Recent efforts have focused on the incorporation of biobased and renewable building blocks. The hydrolytically degradable PSEs are of particular interest, and their degradability is different from that of the typical biodegradable ester linkages that require enzymes for degradation. Furthermore, the degradation products of PSEs include diols and silanols/siloxanes, which are relatively nontoxic. This makes PSE-based materials suitable for short-term and/or single-use applications [30,31]. These characteristics of PSEs are well aligned with the standards and guidelines required for sustainable polymers [32,33,34], and there has been increasing interest in PSEs and PSE-based materials.

In the current review, we will first summarize the synthetic approaches for the PSEs in the literature, followed by the properties and applications of these materials. At the end, we will provide conclusions and a perspective on PSEs. Inevitably, copolymers with other linkages in their main chains will be included in certain cases, but our focus will remain on the PSEs featuring Si-O-C linkages. The bifunctional silyl ether, in which two Si-O-C connections originate from the same silicon atom, is sometimes termed silicon acetal, or silaketal, and will be included without further distinction. Furthermore, selected polymeric materials in which the silyl ether linkage is a relatively minor component by percent composition but plays a significant role with respect to their properties are also covered. The analogous polymers containing nitrogen, such as polysilazanes and organopolysilazanes, are not covered. A recent review article focuses on PSEs synthesized from dihydrosilanes and various dicarbonyls (or hydroxyl carbonyls) [35], and another review focuses on silicon-based PSEs and other phosphorous-based macromolecules as degradable biomedical polymers [36].

## 2. Synthesis of PSEs

There are various known ways of synthesizing PSEs depending on the linkage types and the leaving groups on the silanes. Based on the leaving or reacting groups of silicon, two major types of silanes, disubstituted silanes (R_2_SiX_2_) and dihydrosilanes (R_2_SiH_2_), are commonly used as the silicon source. Silicon sources with more than two reacting groups, e.g., RSiH_3_ and RSi(OMe)_3_, can be employed as well, though they tend to generate branched and crosslinked products. 

### 2.1. From Disubstituted Silanes

Disubstituted silanes (R_2_SiX_2_) are one of the generally employed silicon sources in PSE synthesis. Typically, the Si atom is attached to two alkyl or aromatic groups (R) and two other substituents (X), such as Cl, OPh, OMe, OTf, NHPh, and NEt_2_. More generally, this type of silane also includes compounds with multiple silicon centers in which each Si center is attached to a reactive X group. In these cases, the resulting polymers can be copolymers of PSEs and polysiloxanes. 

#### 2.1.1. R_2_SiX_2_ with Diols

**Scheme 2:**
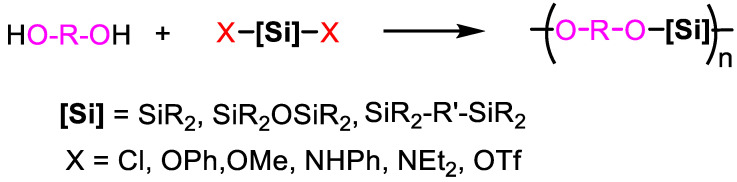
Synthesis of PSEs using [Si]X_2_ with diol.

Among this type of silanes, dichlorosilanes are the most common for the synthesis of PSEs. Similar to the synthesis of silyl ethers, reaction of dichlorosilanes with diols via the conventional condensation polymerization protocol leads to the formation of PSEs (Scheme 2) [37,38,39,40,41,42,43]. However, the use of dichlorosilanes has limitations, as the byproduct HCl requires a base to neutralize it and additional methods for its separation [44]. The hydrolytic sensitivity and corrosiveness of chlorosilanes make them inconvenient for handling and storage. Therefore, chlorosilanes are often replaced with diamino- and dialkoxysilanes as the coupling partners; in particular, diphenoxy- and dianilinosilanes have shown good activity towards the synthesis of PSEs [15,45]. However, low-molecular-weight polymers and high reaction temperatures are the considerable drawbacks of dialkoxysilane polymerization reactions. Besides these commonly used starting materials, a few others, such as X = OTf [46] and NEt_2_ [47], have also been employed as substitutes for silanes.

**Scheme 3:**
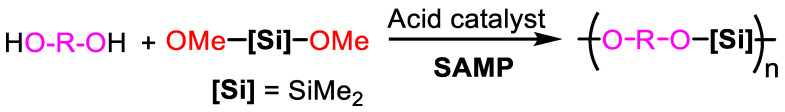
Synthesis of PSEs via SAMP between dialkoxysilane and diol.

In the presence of a Bronsted acid catalyst, such as triflic acid, methanesulfonic acid, or trifluoracetic acid, the reaction of diols and dimethyldimethoxysilane Me_2_Si(OMe)_2_ afforded oligomeric silicon acetals via a step-growth copolymerization reaction which further produced bis-silicon acetals (Scheme 3) [48]. The polymerization of bis-silicon acetals with the elimination of a volatile silicon acetal, dimethoxydimethylsilane, produced final PSEs with molecular weights of up to 26.6 kDa. Since the alkoxylsilane precursors can be viewed as silicon acetals, the reaction has been termed silicon acetal metathesis polymerization (SAMP). This metathesis/exchange strategy and its variations can be useful in introducing silyl ether bonds as crosslinks. 

#### 2.1.2. R_2_SiX_2_ with Bisepoxide and Bisoxetane by Polyaddition 

**Scheme 4:**
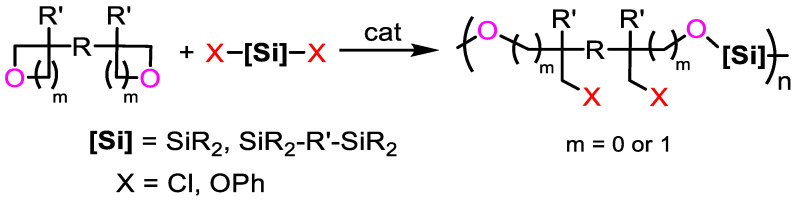
Synthesis of PSEs from bis(epoxide)s and bis(oxetane)s by polyaddition.

Addition reactions of bis(epoxide)s and bis(oxetane)s with dichlorosilanes yield PSEs in high yields (95–99%) (Scheme 4). The reactions are catalyzed by quaternary onium salts, such as tetrabutylammonium chloride (TBAC), tetrabutylammonium bromide (TBAB), and tetrabutylphosphonium chloride (TBPC). The molecular weights can reach 53 kDa under suitable conditions using aromatic and lipophilic solvents, including chlorobenzene, anisole, and toluene [29,49,50,51,52]. In addition to dichlorosilanes, diphenoxylsilanes can be used under similar conditions [53]. Notably, the methylene chloride functional group on the side chain in the resulting PSEs provides further opportunities for functionalization.

### 2.2. From Dihydrosilanes

In developing new methodologies to produce PSEs in a more sustainable manner, hydrosilanes have emerged as suitable silylating reagents. Moisture stability, ease of handling, and elimination of unwanted byproducts make them optimal replacements for chlorosilanes. Besides the typical dihydrosilanes (R_2_SiH_2_), bis(hydrosilane)s, such as 1,4-bis(dimethylsilyl)benzene (BDMSB), can be utilized for the reaction [54]. The introduction of additional groups (aromatic, alkyl, and oxygen groups) between the two silicon atoms of bis(hydrosilane)s widens the scope of accessible structures and properties [55,56].

Two main pathways exist for the formation of PSEs from dihydrosilanes: hydrosilylation of carbonyl compounds and dehydrogenative cross coupling with alcohols. The former reaction generates no byproducts, and the latter one has the sole byproduct of H_2_(g); thus, they are highly atom economical. Both processes require catalysts, the majority of which are traditionally based on precious metals, such as Pt, Pd, Ru, and Rh [57,58,59]. However, the high cost, low abundance of such metals, and possible side reactions are the main drawbacks of these methods [60]. More recent studies have focused on cheaper alternative earth-abundant base metals, such as Fe [61,62,63], Mn [64,65], Zn [66], etc., and alkali metals [67]. Some catalysts are active for both types of reactions, thereby broadening the types of substrates suitable for PSE formation. 

#### 2.2.1. Hydrosilylation Polymerization with Dicarbonyls

**Scheme 5:**
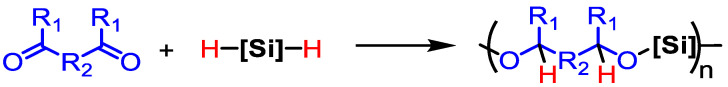
Hydrosilylation polymerization of dicarbonyl substrates with silanes.

Hydrosilylation of carbonyl compounds is used for the reduction of unsaturated organic compounds, involving the addition of silicon hydrides across unsaturated (carbonyl) bonds. Reactions of dihydrosilanes with dicarbonyls through hydrosilylation polymerization have been employed for the synthesis of PSEs (Scheme 5). A number of metal-based catalysts can be utilized in the reactions [68,69,70]. Recently, metal-free synthesis of PSEs has been described through Lewis acid B(C_6_F_5_)_3_-catalyzed step-growth polymerization of α-diketones and BDMSB [71]. A number of PSEs with various functionalized polymer backbones and high molar masses were synthesized. 

Interestingly, using a hydrosilylation polymerization approach, Zhou’s group developed a CuH chiral bis(phosphine)-based catalytic system for the synthesis of optically active PSEs with main-chain chirality [72]. A variety of aromatic and heteroaromatic diketones could be employed with high molecular weights and excellent yields of up to 98%. The enantioselectivities were up to 99% *ee*. Furthermore, the catalysts were highly effective, requiring a low catalyst loading (0.5 mol%) under mild conditions. Thermal analysis indicated that these enantiomerically enriched PSEs exhibit good thermal properties and may be promising in applications such as chiral separation. 

#### 2.2.2. Dehydrogenative Cross-Coupling Polycondensation with Diols

**Scheme 6:**
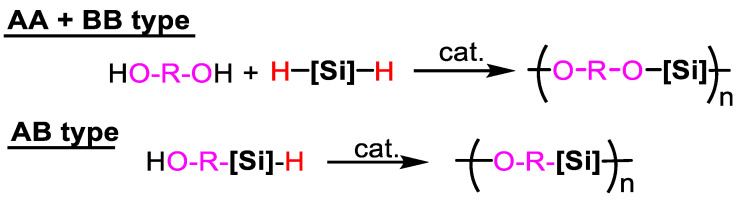
Synthesis of PSEs with AA + BB- and AB-type monomers.

Synthesis of PSEs from diols and hydrosilanes has been another effective protocol due to its high atom economy [27,54,73]. The dehydrogenative cross coupling can take several routes (Scheme 6), demonstrating the versatility of this method for the synthesis of PSEs. The AA + BB-type polycondensation reaction between a dihydrosilane and a diol is commonly used, since the two monomers can be independently varied, allowing great structural diversity in PSEs. On the other hand, the AB-type monomers containing both OH and SiH groups offer precise stoichiometry control of the two functionalities. 

**Dehydrogenative Cross Coupling of AA + BB-Type Monomers**. The AA + BB approach is probably the most common, and only a few selected examples are discussed here. Partially biobased PSEs were synthesized via dehydrogenative cross-coupling polymerization catalyzed by an air-stable anionic iridium complex bearing a functional bipyridonate ligand [55]. Moderate to high yields of polymers with *M*_n_ values of up to 43.8 kDa were obtained. Diol monomers derived from biobased levulinic acid and succinic acid were applied in the synthesis of PSEs. Vijjamarri et al. synthesized several degradable PSEs from biobased 1,4:3,6-dianhydrohexitols (such as isosorbide and isomannide) and commercially available hydrosilanes by a manganese-catalyzed dehydrogenative cross-coupling reaction [74]. PSEs with *M*_n_ values of up to 17 kDa were obtained from isosorbide and diphenylsilane. 

Morris et al. found that dehydrogenative cross coupling of silanes and diols can be mediated by heavier alkaline earth catalysts [75]. With a precatalyst [Ba{N(SiMe_3_)_2_}_2_·(THF)_2_] in THF, ferrocene-containing PSEs with ferrocene pendent to or as a constituent of the main polymer chain were prepared from Fe(C_5_H_4_SiPhH_2_)_2_ with diols 1,4-(HOCH_2_)_2_-(C_6_H_4_) and 1,4-(CHMeOH)_2_(C_6_H_4_), respectively. The resultant polymeric materials had *M*_n_ values >20 kDa. Importantly, the Fe centers displayed reversible redox behavior, and thermal analysis showed that they could be promising precursors to magnetic ceramic materials.

Zhou et al. used Co- and Ir-based catalysts to produce optically active PSEs from asymmetric dehydrocoupling of chiral silanes and diols [76]. The chirality of PSEs can be used for chiral catalysis, chiral column packing, and nonlinear optics. Rhodium-based catalytic systems and other monomers, such as prochiral silanes, were also tested to make similar structures with a high molecular weight and good thermal stability.

The B(C_6_F_5_)_3_-catalyzed dehydrocondensation of bis-phenols with dihydrosilanes and siloxanes provides ready access to the polyaryloxysilanes (PASs) [77]. Sterically protected PAS derivatives with *M*_n_ values of up to 51.8 kDa were obtained from hindered phenol or silane substrates with low levels of boron catalysts (as low as 0.04 mol%). Phenol-capped macromonomers derived from polycarbonate and poly-2,6-dimethylphenylene oxide (PPO) were readily coupled in the reaction, affording siloxane copolymers. Because of the Lewis acidity of B(C_6_F_5_)_3_, base functionality, such as amines in either monomers or solvents, was not tolerated in the reaction. 

**Dehydrogenative Cross Coupling of AB-Type Monomers**. The AB-type monomer can be advantageous, since there is only one monomer to measure out which reacts with itself. This guarantees a 1:1 ratio of two functional groups, which is important for step-growth polymerization to reach high molecular weights. However, such monomers often require multistep synthetic procedures for construction. Cheng et al. utilized AB-type monomers derived from biorenewable feedstock and obtained a series of PSEs with relatively high *M*_n_ values [78]. These polymers showed good thermal stability, biocompatibility, and degradability. Zhou’s group reported an iridium-catalyzed dehydrocoupling polymerization of AB-type silyl alcohol monomers [56]. PSEs with *M*_n_ values of up to 92.7 kDa were obtained with moderate to high yields.

#### 2.2.3. Polymerization with Bifunctional Monomers 

**Scheme 7:**
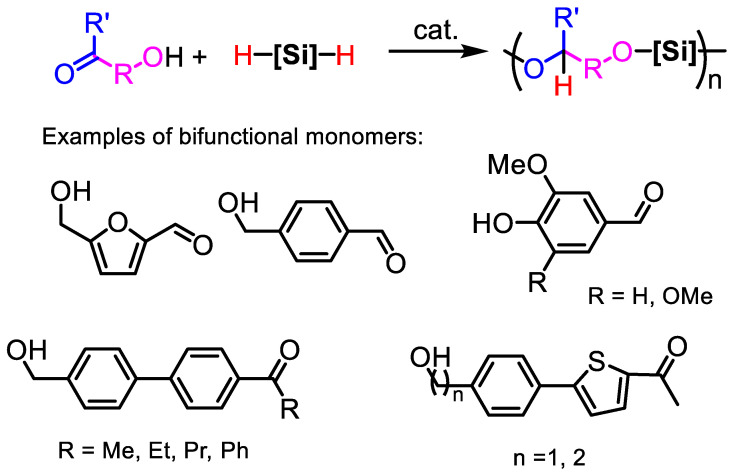
Dihydrosilane reaction with bifunctional monomers to form PSEs.

Hydroxyl carbonyl compounds are widely available; however, it can be challenging to directly utilize them as monomers in polymer synthesis because of the presence of two different functional groups. A few catalytic systems have shown activity for the hydrosilylation of carbonyls and dehydrogenative cross coupling of alcohols with hydrosilanes; thus, they can be employed in the polymerization of hydroxyl carbonyl compounds with dihydrosilanes (Scheme 7). Earlier work with a Pd/C catalyst indicated the potential of such an approach, which opens doors for different uses of silanes and PSEs due to their ability to react with two different functional groups [59]. Vijjamarri et al. reported the polymerization of a series of hydroxyl carbonyl compounds catalyzed by a manganese salen compound [79]. In particular, 5-hydroxymethyl furfural (HMF), a biobased platform molecule, was polymerized with Ph_2_SiH_2_ to obtain PSEs with a decent yield (74%) and *M*_n_ values of up to 8.0 kDa [80]. Li et al. used a dual catalytic system of a Zn hydride and B(C_6_F_5_)_3_ for the polymerization of HMF with dihydrosilanes [81]. B(C_6_F_5_)_3_ alone would lead to additional side reactions. More recently, two Pt carbene complexes were reported for the effective synthesis of PSEs from HMF, as well as other biobased monomers, such as vanillin and syringaldehyde [82]. By using Cu-H catalysts supported with chiral phosphine ligands, a series of chiral PSEs were synthesized from dihydrosilanes and bifunctional hydroxyketones through asymmetric hydrosilylation/dehydrocoupling polymerization [83]. The molecular weights were high, reaching up to 36.8 kDa, with excellent enantioselectivity (ee% values of up to 94%). In a related advance, silyl ether–carbosilane copolymers were obtained from BDMSB and unsaturated ketones by B(C_6_F_5_)_3_-catalyzed hydrosilylation polymerization of both C=O and C=C functionalities [84].

#### 2.2.4. Polymerization with Diepoxides

**Scheme 8:**
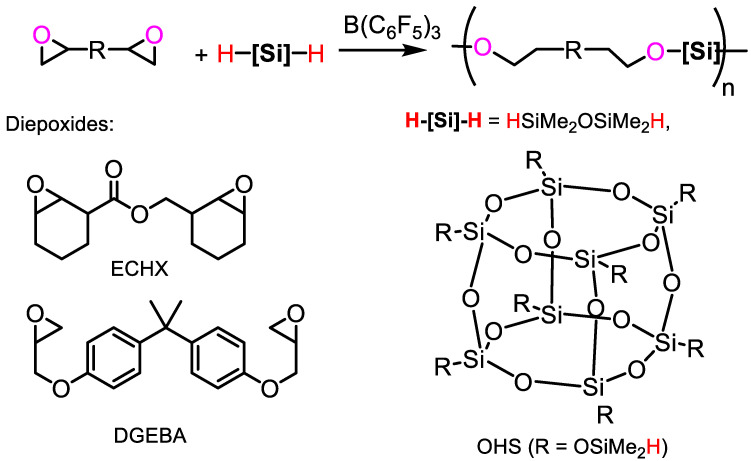
Oxylation of diepoxides with hydrosilanes catalyzed by B(C_6_F_5_)_3_.

Zhang et al. have shown that the oxysilylation reaction of diepoxides and hydrosilanes is catalyzed by B(C_6_F_5_)_3_ at ambient temperature by ring opening of the epoxides (Scheme 8) [85]. The diepoxides reacted with dihydrosilanes such as tetramethyldisiloxane (TMDS) to form linear PSEs with *M*_n_ values in a range of 1–20 kDa. The strategy has been employed to synthesize self-reinforced epoxy resin nanocomposites using various diepoxides and hydrosilanes with multiple Si-H bonds, such as octafunctional cubic silsesquioxane [HMe_2_SiOSiO_1.5_]_8_ (OHS), tetramethylcyclotetrasiloxane [CH_3_SiHO]_4_, and pentamethylcyclopentasiloxane [CH_3_SiHO]_5_. Diepoxides with simple structures reacted rapidly, while the increasing complexity of the diepoxides resulted in decreased reaction rates. Oxysilylation of diepoxides with OHS typically formed gels before full completion of the reactions due to high crosslink densities.

#### 2.2.5. Polymerization with Dialkylether of Bis-Phenols 

**Scheme 9:**
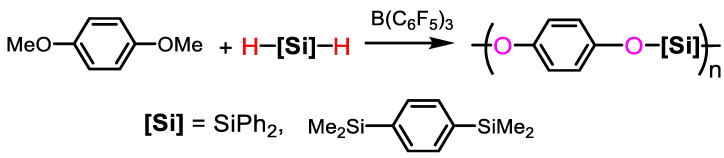
Polyaryloxysilanes obtained from dihydrosilanes and dialkylethers catalyzed by B(C_6_F_5_)_3_.

Cella et al. noted that B(C_6_F_5_)_3_ catalyzed the polycondensation of dialkylethers of bis-phenols with dihydrosilanes (Scheme 9) [77]. The reaction byproduct was a hydrocarbon instead of hydrogen gas with bis-phenols. This variant can be useful when the dialkylether is more readily soluble than the bis-phenol in the reaction solvent. 

### 2.3. Polyaddtion of Cyclopolysilanes and Cyclodisilanes 

**Scheme 10:**
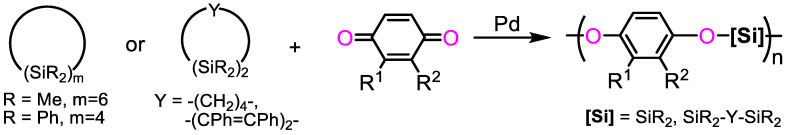
Polyaddition of cyclopolysilane and cyclodisilane with quinone derivatives.

Reddy et al. reported a Pd-catalyzed ring-opening polyaddition strategy for the synthesis of PSEs [86]. Cyclopolysilanes and cyclodisilanes with Si-Si bonds were allowed to react with the quinone derivatives (Scheme 10) to afford PSEs with *M*_n_ values of up to 82 kDa; however, the dispersity was high (*Đ* values of up to 11), suggesting that the polyaddition process was poorly controlled. The method was also limited in scope, as only quinone derivatives were shown to be active. 

### 2.4. Rearrangement of Poly(acyl silane)s 

**Scheme 11:**
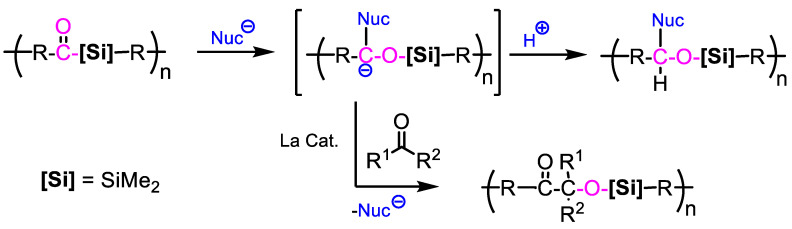
Rearrangement of poly(acyl silane)s to PSEs.

A novel method has been reported recently in which the skeletal rearrangement of poly(acyl silane)s leads to the formation of PSEs via the anionic 1,2-Brook rearrangement (Scheme 11) [87]. The acyl silane functional group was incorporated into polymer backbones using acyclic diene metathesis (ADMET) copolymerization of acyl silane-containing diene with another ADMET monomer such as 1,9-decadiene. The rearrangement was initiated by a strong nucleophile such as PhLi, yielding PSEs with the nucleophile attached to the chain. With a lanthanide catalyst, the rearrangement intermediate was intercepted by a ketone, leading to the Si-O-C-containing polymers bearing a quaternary stereogenic center in the rearranged backbone. This represents a powerful approach for an array of functional PSEs inaccessible by conventional means. 

### 2.5. Other Synthetic Pathways for PSEs

**Scheme 12:**
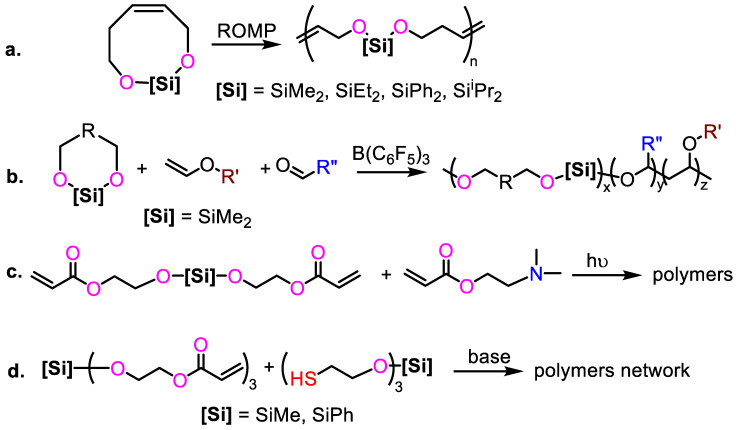
Various polymerizations and copolymerizations of Si-O-C-containing monomers.

In the above approaches, the Si-O-C bond is generated during the polymerization process. Alternatively, the silyl ether bond could be pre-formed in various types of monomers from which copolymers of PSEs can be prepared. Johnson and coworkers synthesized PSEs by using ring-opening metathesis polymerization (ROMP) of alkenes [88,89,90]. The silyl ether-based cyclic alkenes were copolymerized with norbornene derivatives and norbornene-terminated macromonomers to form PSEs (Scheme 12a). Hada et al. developed a B(C_6_F_5_)_3_-catalyzed cationic terpolymerization of cyclic silyl ethers with vinyl ether and aldehydes to silyl ether polymers with pseudo-periodic ABC-type sequences (Scheme 12b) [91]. Parrot et al. reported the radical polymerization of dimethylaminoethyl acrylate with Si-O-C-containing bis(acrylate)s crosslinkers in the presence of UV light or other radical forming reagents (Scheme 12c) [92]. Bunton et al. synthesized a degradable, crosslinked polymer network from silyl ether-containing trithiols and triacrylates using base-catalyzed thiol Michel addition reactions (Scheme 12d) [93]. Such approaches take advantage of the vast toolkit available for other functional groups and may offer better control of the polymerization process.

## 3. Properties and Applications of PSEs

As mentioned earlier, PSEs display excellent thermal stabilities and low-temperature flexibilities, along with tunable degradability under both acidic and basic conditions. Indeed, the hydrolytic degradability of PSEs is one of the main reasons for recent interest in PSE-based materials. On the one hand, the degradation of PSEs requires no microbes and occurs more readily under environmental conditions. On the other hand, the controllable degradation under acidic or basic conditions can be utilized to construct stimuli-responsive materials. In this section, we discuss the thermal properties of representative linear PSEs and present the hydrolytic degradation behavior of selected examples. This is followed by a discussion of the application of PSEs and PSE-containing materials in related fields, most of which is based on the hydrolytic degradability of the Si-O-C linkage.

### 3.1. Thermal Properties and Hydrolytic Degradability 

The thermal properties, including the thermal degradation temperatures (T_−5%_ and T_−50%_, weight loss at 5% and 50%, respectively) and the glass transition temperatures (T_g_), are usually determined by using TGA and DSC techniques under a nitrogen atmosphere. Results for selected linear PSEs are summarized in Table 1. PSEs, generally, are thermally stable, with the decomposition onset temperature typically above 350 °C. The glass transition temperatures cover a wide range from −95 to 120 °C, depending on the composition of the polymers. 

Because of their rigid bicyclic structure, isohexide-derived PSEs possessed high thermal stability with T_−5%_ values of 347–446 °C and T_g_ values of 42–120 °C [74]. Structure–property analysis suggested that steric bulk and molecular weight have a significant influence in determining the thermal properties of synthesized polymers. When rigid isosorbide was combined with a bulky hydrosilane containing a naphthyl group, T_g_ values of up to 120 °C were observed (Table 1, **P5**). Importantly, these polymers were degraded effectively to small molecules under acidic and basic hydrolysis conditions within a few days.

Poly(siloxyethylene glycol) (PSEG) was synthesized through polycondensation reactions between oligosiloxane and oligo(ethylene glycol) with a monomodal molecular weight distribution in the range of 3.5–17.7 kDa [47]. The silicon content of PSEG showed a significant influence on its properties. The T_g_ of PSEG decreased from −53 to −100 °C with increasing Si content, and PSEG with a <15 wt % silicon content became water soluble. PSEG with a disiloxane linkage showed higher hydrolytic stability in water than PSEG with a monosiloxane linkage, though both hydrolyzed within hours. 

Zhou and coworkers synthesized PSEs via iridium-catalyzed dehydrocoupling polymerization, and the thermal properties of these polymers were investigated. For chainlike polymers, T_−50%_ values were around 470 °C, indicating good thermal stabilities similar to those of PSEs synthesized via hydrosilylation. The glass transition temperatures were low (around −80 °C), and polymers containing an aromatic backbone showed higher T_g_ values than those containing aliphatic backbones. This suggested that incorporating aromatic groups in the main chain of the polymer might increase the barrier of longitudinal motion of the polymer chains.

Diols derived from palm oil and soy oil were also employed for synthesizing new PSEs [94]. The TGA study illustrated that these PSEs underwent two-step degradation. The initial degradation began at a temperature below 250 °C, which was attributed to the degradation of the long alkyl carboxyl side chain, and the second stage of degradation was the cleavage of silyl ether linkages in the polymeric backbone. The soy oil-based PSE was observed to be more stable than the palm oil-based PSE, which could be explained by the fact that soy oil-based diol possesses more C=C double bonds than palm oil-based diol, resulting in strong intermolecular interactions. The DSC data also revealed that soy oil-based PSEs had a slightly higher T_g_ (34.2 °C) than palm oil-based PSEs (31.6 °C). 

In a structure–property relationship investigation of PSEs [71], the aromatic systems based on benzil and BDMSB showed high thermal stability, as increased T_−5%_ values (~415 °C) were observed when compared to traditional siloxanes (Table 1, entries 13 vs. 18). Depending on the side-chain groups (derived from the diketone) and the backbone linker unit (derived from the bis(silane)), the T_g_ could be tuned from −30 to 80 °C. Interestingly, a wide range of such PSE materials were semicrystalline; for example, the alkyl-substituted hexane-3,4-dione derivative (Table 1, **P16**) was observed to have sharp transitions, with a T_c_ of 30 °C and a T_m_ of 120 °C, while the phenylene-bridged PSE with the benzil-based system (**P13**) showed a T_c_ of 135 °C and a T_m_ of 175 °C. When the freely rotating phenyl rings were replaced with a rigid phenanthrene unit, the T_g_ was observed to increase, but an increase in the T_c_ or T_m_ was not observed in the measurement window of crystallinity, which was attributed to the stereochemically random nature of the backbone. The degradability of the silphenylene copolymers (**P13**–**15**) was found to be stable against alcoholysis after an extended period, while the siloxane-based copolymer showed a 2-fold molecular weight decrease. In the presence of triethylamine, both the silphenylene copolymer (**P13**) and the siloxane copolymer (**P18**) showed no apparent molecular weight change. Under acid-catalyzed hydrolysis, complete degradation was observed within 12 h for **P18** and over 5 days for **P13**. The difference could be attributed to the steric hindrance and electronic nature of the Si–O bonds. The tunability of PSE properties, such as acid-promoted degradation, is an important feature for the construction of responsive Si-based polymeric materials.

Wooley’s group prepared a series of copolymers containing both the silyl ether and the ester linkages in the main chains by reacting poly(*Ꜫ*-caprolactone) (PCL)-based macrodiols with a silicon precursor, BDMSB [95]. The resulting copolymers showed a two-stage degradation behavior: the silyl ether linkages readily broke down when treated with acids to regenerate the PCL blocks of intermediate molecular weight, while the ester linkages needed a stronger acid and longer time for degradation to continue the transition from polymeric materials to small molecules. This stepwise degradation profile complements the existing repertoire of degradation modes. Modulation of the degradation properties for specific applications is expected to be conveniently accomplished via adjustments in the chemical composition of the silyl linker, the number of types of copolymerized monomers, and/or the selection of macromonomers with various molecular weights. 

### 3.2. Applications in Materials Science

Hawker’s group used the B(C_6_F_5_)_3_-catalyzed hydrosilylation of *α*-diketones by linear siloxanes as a strategy for rapid, efficient vulcanization of silicones [96]. In this approach, catalytic crosslinking of poly(dimethyl-co-methylhydro)siloxane (PMHS), commercially available with a range of molecular weights and hydrosilane contents, with readily available *α*-diketones, led to the formation of network polymers bearing Si-O-C linkages. Such materials are less stable hydrolytically when compared to traditional Pt-cured silicones. As an example, samples of PMHS (6 kDa, 8% Si–H) crosslinked with either 3,4-hexanedione (by boron catalysis) or divinyltetramethyldisiloxane (by Pt catalysis) were studied under different conditions. While neither sample showed appreciable mass loss within 2 weeks when exposed to methanol or triethylamine, the B(C_6_F_5_)_3_ sample dissolved completely within 24 h when immersed in HCl solution (1 mM in chloroform); over that same period, the Pt sample remained intact. The hydrolysis products were poly(dimethyl-co-methylhydroxy)siloxane and 3,4-hexanediol. The siloxane backbone itself could degrade after a prolonged time: after 1 month in the 1 mM HCl solution, the Pt samples eventually dissolved. The relative stability is attributed to the poor swelling of the network with the solvent, reducing the exposure of the siloxane backbone to acid relative to the fully dissolved chains. 

The oxysilylation reaction of diepoxides with hydrosilanes was utilized for making self-reinforced epoxy resin nanocomposites [85]. The resulting polymeric gels started to decompose at ∼200 °C in air, which was attributed to an exothermic reaction and likely oxidation of residual Si–H groups. The 1:1 DGEBA/OHS system (see Scheme 8) offered higher decomposition temperatures (~300 °C), likely because of the phenyl component in the backbone. The 1:1 ECHX/OHS system showed two main mass losses: one from 200 to 270 °C and the other from 400 to 440 °C. The first mass loss may be associated with oxidation of residual Si–H groups. The second mass loss was similar to other systems that involved oxidation of carbon species (≥350 °C). Notably, these polymeric gels demonstrated good water stability, resisting boiling water for several hours, even when some residual Si-H bonds were still present.

Zhang et al. incorporated Si-O-C linkages into the main chains of polyurethane thermosets [97]. This allowed polymer degradation in response to weak acids such as acetic acid, and the alkyl substituents of the silicon showed a significant influence on the degradation rates, which could be tuned from hours to months for complete degradation. In combination with the excellent mechanical, physical, and chemical properties of polyurethanes, such degradation-controllable polyurethane thermosets are promising for green polymer product manufacture.

Johnson and coworkers developed degradable thermosets based on silyl ether linkages [98]. Cyclic alkenes containing Si-O-C bonds were introduced as comonomers in the industrial thermoset polydicyclopentadiene (pDCPD) system. When the incorporation of the silyl ether-containing cyclic alkene comonomers was ~10% v/v, the resulting thermosets maintained many of the properties of the native materials, such as the Young’s modulus, % strain at break, toughness, and thermal stability. Due to the cleavability of the silyl ether linkage, such thermosets could chemically degrade into soluble and recyclable products under mild conditions. Interestingly, the location of the cleavable bonds was critical for the degradability, as the materials with the cleavable silyl ether bonds in the crosslinks were not degradable under similar conditions. By introducing 10% v/v strand-cleaving *crosslinkers* (SCCs) that provided cleavable pDCPD network junctions, the pDCPD samples regained degradability under mild conditions, while the T_g_ was 48 °C higher than that of an analogous degradable pDCPD made using cleavable *comonomers* and comparable to that of the virgin pDCPD [99]. Thus, this SCC strategy does not require a complete replacement of existing material components but may enable access to deconstructable materials with improved thermomechanical performance. In addition to the cleavage reactions, the Si-O bond can also undergo exchange reactions, so its incorporation into a thermoset may generate covalent adaptable networks that are not only deconstructable, but also reprocessible [100]. Thus, the pDCPD thermosets installed with the silyl ether linkages by copolymerization behaved as covalent adaptable networks in the presence of an organic acid as a catalyst, displaying temperature- and time-dependent stress relaxation as a function of their substituents and allowing healing and remolding of the materials. Incorporation of the silyl ether bond into the polyethylene backbone by ROMP copolymerization of silyl ether-based cyclic alkenes (see Scheme 12a) and cyclooctene followed by hydrogenation afforded high-density polyethylene-like materials that are thermally robust yet chemically deconstructable and recyclable [101]. 

**Scheme 13:**
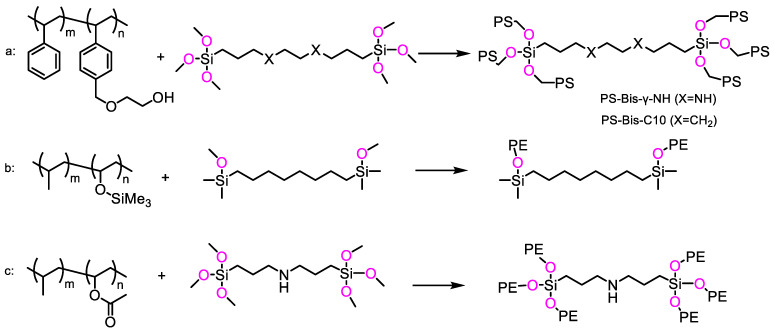
Vitrimers based on silyl ether crosslinkers (PS: polystyrene; PE: polyethylene).

Guan and coworkers crosslinked a pendant hydroxyl group-functionalized styrene-based copolymer with bis-alkoxysilanes [102]. By incorporating silyl ether linkages through the exchange reaction into covalently crosslinked polystyrene networks, dynamic vitrimers with malleability and reprocessibility were obtained. Two crosslinkers, one with amino neighboring groups, and the other without, afforded two network materials, PS-Bis-γ-NH and PS-Bis-C10 (Scheme 13a). Both displayed very high thermal stability, as negligible thermal degradation was observed at temperatures below 300 °C for PS-Bis-γ-NH and 350 °C for PS-Bis-C10. The degradation onset temperature was ~100 °C higher than that for other known vitrimers, allowing material processing and applications in a broader temperature range. A dramatic difference in the temperature-dependent stress–relaxation behavior of the two materials was observed, which was correlated with the silyl ether exchange-rate accelerating effect of the neighboring amino moiety in PS-Bis-γ-NH. In an alternative strategy for silyl ether exchange, Guan’s group discovered that silyl ether groups can directly exchange (i.e., silyl ether metathesis) without the need for free hydroxyl groups (Scheme 13b) in the presence of an acidic catalyst [103]. The resulting polyethylene-based materials showed exceptional thermal stability, with minimal loss below 400 °C and T_−5%_ at 427 °C, representing some of the most thermally stable vitrimers reported so far. The lack of free hydroxyls and the robustness of silyl ether moieties were believed to be responsible for the high thermal stability. 

In a similar work, the thermally stable silyl ether crosslinks were incorporated into an industrial poly(ethylene-vinyl acetate) (EVA) product to form EVA vitrimers (Scheme 13c) [104]. This led to an improvement in the Young’s modulus and tensile strength by four and two times, respectively. Increasing crosslinking density also led to increased creep resistance. Furthermore, the EVA vitrimers could be recycled and reprocessed multiple times without much change in mechanical properties, such as the Young’s modulus, tensile strength, and elongation at break.

### 3.3. Applications in Biomedicine

Polymeric nanoparticles are some of the commonly used carriers for drug delivery because the polymers can be functionalized to target specific cell receptors and release a specific drug or other compound with suitable triggers [105]. Due to their hydrolytic degradation properties and ease of functionalization, PSEs are ideal candidates for making nanoparticles for drug delivery. 

**Scheme 14:**
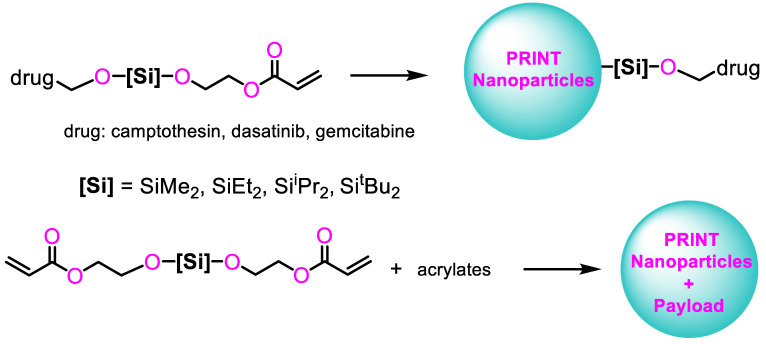
PRINT nanoparticles containing silyl ethers as linkers and crosslinkers.

Parrott et al. used silyl ether bonds to link drugs to the surface of nanoparticles made through a particle fabrication process called PRINT (particle replication in nonwetting templates) [106]. The release of the drug under acidic conditions was successful, and the rate of release was modified by changing the substituents on the silicon (Scheme 14, top). For **[Si]** = Si^t^Bu_2_, the system showed a 100% release of drugs after 25 h at pH 5 and 7. Although the silyl ether bond only serves as a linker and the polymers are technically not PSEs, this work illustrated the potential of PSEs as prodrugs and drug carriers. In a related work, a series of silyl ether-containing diacrylates were employed as crosslinkers (Scheme 14, bottom) [92]. The resulting polymeric materials were made into nanoparticles with controlled sizes and shapes using the PRINT technique. The incorporation of the Si-O-C linkage imparted degradability to the PRINT nanoparticles. After rhodamine, as a probe, was loaded, the release of rhodamine was shown to be best at a pH of 5, with all three crosslinkers having different substituents. Although the desired release (>80%) took 4 days, which was not optimal, this demonstrated promise for PSE-based nanoparticles, with their release and degradation being tunable.

**Scheme 15:**
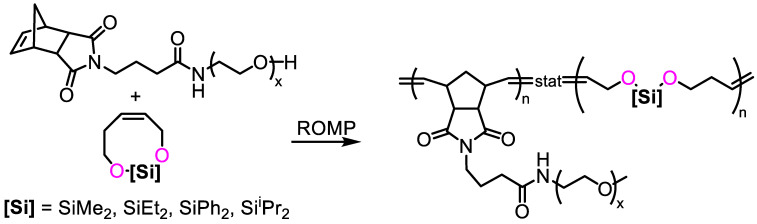
Bottlebrush polymers with degradable silyl ether in the backbone.

Johnson’s group described a class of bifunctional silyl ether-based cyclic olefins that copolymerize efficiently with norbornene-based (macro)monomers to provide copolymers with controlled sizes, narrow molar mass distributions, and varied architectures, including linear, bottlebrush, and brush-arm star copolymers [88]. As an example, a bottlebrush polymer design with PEG side chains is shown in Scheme 15. The presence of the silyl ether segment in the backbone enabled tunable degradation kinetics of the materials and the manipulation of the in vivo biodistribution and clearance profiles. Specifically, significant degradation was observed at mildly acidic pH values (5.5 and 6.5), aided by the materials’ high solubility in aqueous media (>25 mg/mL). The relative extents of degradation followed the expected reactivity trends of the silyl ether functional groups (in the order of Me, Et, Ph, and ^i^Pr, from fastest to slowest), which allowed tuning of the degradation kinetics over several orders of magnitude at physiologically relevant pH values. As expected, the degradation began from the more accessible bottlebrush backbone chain ends and proceeded inwardly. These samples were shown to be biocompatible, as they showed minimal cytotoxicity at the 1 mg/mL concentration level, and samples with ^i^Pr and Ph substituents showed increased cell uptake. Finally, such PEG-based bottlebrush polymers with degradable backbones were shown to display long-term in vivo biodistribution and clearance profiles that were distinct from those of their counterparts without the silyl ether segment in the backbone, as significantly lower amounts of accumulation in the liver and spleen were observed after 3 weeks of administration in mice. 

## 4. Conclusions and Perspective

In conclusion, poly(silyl ether)s (PSEs) are an interesting class of polymeric materials. A variety of synthetic methods have been developed, with the majority of them starting from hydrosilanes that are easy to handle and produce no or few byproducts. PSEs with diverse backbone structures have been obtained, and the silyl ether linkages can be incorporated into other polymeric materials as copolymers, crosslinkers, or side chains. In addition to the thermal stability and a broad range of glass transition temperatures available for PSEs, the hydrolytic degradability of the silyl ether bonds is a distinct feature that is being exploited in the application of PSEs, particularly in their use as drug carriers in the biomedical fields. 

The current renewed interest in PSEs is in part driven by the demand for sustainability, in particular, the incorporation of biobased monomers into various polymeric materials [107]. This consideration is highly relevant for PSEs: compared to petroleum-derived feedstocks, biomasses are rich in oxygen content, the removal of which often requires challenging deoxygenation processes. With PSEs, part of the oxygen content of biomass can be directly incorporated without excessive deoxygenation. In fact, much of the recent research on PSEs has been carried out with a range of biobased starting materials, mostly diols, along with dicarbonyls and bifunctional hydroxycarbonyls. The direct utilization of 5-hydroxymethyl furfural (HMF), a readily available biobased bifunctional platform molecule, as a comonomer in PSE synthesis, and the attainment of high T_g_ values with isohexide monomers are some of the notable advances in the field. Given the large number of biobased monomers and their derivatives, there are unlimited possibilities for new materials with tailored properties. In a related consideration for sustainability, new catalysts derived from earth-abundant, inexpensive, low-toxicity, and biocompatible metals, such as Fe, Mn, Cu, and Zn, are being designed and employed in PSE synthesis.

So far, PSE-based materials have mostly utilized the hydrolytic degradability of the silyl ether linkages. In order to broaden the scope of their application, it can be envisioned that units responsive to other stimuli, such as light, temperature, and redox environment, could be incorporated into the materials. The AA + BB-type synthetic methods for PSEs are inherently flexible, as one monomer can be modulated independently of the other, while maintaining the silyl ether linkages in the final products; thus, they are likely the first methods of choice for targeted PSEs. Furthermore, other, more complex polymer architectures, such as hyperbranching, crosslinking, and polymer brushes, may be introduced in addition to the typical linear ones. In these cases, the silyl ether linkages can be easily introduced post-polymerization by exchange/metathesis reactions with free hydroxyl, methoxy, and acetate groups or by simple polymerization/copolymerization with a silyl ether-functionalized monomer. Clearly, PSEs are highly versatile materials, and more research is needed to fully achieve and extend their potential.

## Figures and Tables

**Table 1:** Thermal properties of selected linear PSEs.

PSE	R	[Si]	*M*_n_ (*Đ*)	T_g_	T_m_/T_c_	T_−5%_	T_−50%_	Ref.
	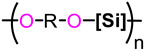							
**P1**	-CH_2_C_6_H_4_CH_2_-	SiPh_2_	9.2 (2.0)	22				[79]
**P2**	-biphenyl-	SiPh_2_	7.2	101				[79]
**P3**	-isosorbide-	SiPh_2_	17.0 (2.08)	85		410	498	[74]
**P4**	-isosorbide-	SiPhMe	13.3 (1.92)	43		397	486	[74]
**P5**	-isosorbide-	SiPhNp	14.0 (2.00)	120		446	509	[74]
**P6**	-isomannide-	SiPh_2_	13.0 (2.25)	76		384	494	[74]
**P7**	-isomannide-	SiPhMe	6.1 (2.05)	42		367	484	[74]
**P8**	-isomannide-	SiPhNp	9.5 (1.56)	116		432	505	[74]
**P9**	-BHMF-	SiPh_2_	4.3 (1.33)	9.8			445	[80]
**P10**	-BHMF-	SiPhMe	3.2 (2.22)	3.5			423	[80]
**P11**	-BHMF-	SiPhNp	2.1 (1.24)	27.8			473	[80]
**P12**	-difuran-	SiPh_2_	6.4 (1.69)	2.5			422	[80]
**P13**	-(CHPh)_2_-	SiMe_2_C_6_H_4_SiMe_2_	55.0 (1.8)	40	175/135	415		[71]
**P14**	-(CHC_6_H_4_Br)_2_-	SiMe_2_C_6_H_4_SiMe_2_	48.2 (1.8)	80	120/-	380		[71]
**P15**	-(CHC_6_H_4_Me)_2_-	SiMe_2_C_6_H_4_SiMe_2_	14.1 (2.1)	−2		195		[71]
**P16**	-(CHEt)_2_-	SiMe_2_C_6_H_4_SiMe_2_	27.3 (2.7)	−25	120/30	310		[71]
**P17**	-2,2′-biphenyl-	SiMe_2_C_6_H_4_SiMe_2_	20.7 (1.7)	70		335		[71]
**P18**	-(CHPh)_2_-	SiMe_2_OS+iMe_2_	7.0 (1.5)	−15	130/95	330		[71]
**P19**	-(CH_2_)_6_-	SiMe_2_	3.4 (1.8)	−46	43/-		175	[48]
**P20**	-(CH_2_)_8_-	SiMe_2_	7.9 (1.1)	−52	57/-		178	[48]
**P21**	-(CH_2_)_9_-	SiMe_2_	9.4 (1.2)	−53	72/-		184	[48]
**P22**	-(CH_2_)_10_-	SiMe_2_	8.3 (1.4)	n.o.	72/-		202	[48]
**P23**	-(CH_2_)_2_OC_6_H_4_O(CH_2_)_2_-	SiMe_2_	6.8 (3.3)	−47	105/-		232	[48]
**P24**	-(CH_2_CH_2_O)CH_2_CH_2_-	SiMe_2_	14.0 (1.5)	−81				[47]
**P25**	-(CH_2_CH_2_O)_6_CH_2_CH_2_-	SiMe_2_	11.8 (1.4)	−72	−30/-			[47]
**P26**	-(CH_2_CH_2_O)_12_CH_2_CH_2_-	SiMe_2_	3.7 (1.2)	−64	17/-			[47]
**P27**	-(CH_2_CH_2_O)CH_2_CH_2_-	SiMe_2_OSiMe_2_	10.8 (1.3)	−95				[47]
	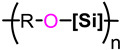							
**P28**	-(CH_2_)_9_-	SiMe_2_	92.7 (3.7)	−76		422	471	[56]
**P29**	-(CH_2_)_10_-	SiMe_2_	25.5 (2.6)	n.o.		350	466	[56]
**P30**	-(CH_2_)_11_-	SiMe_2_	46.9 (2.9)	−83		421	469	[56]
**P31**	-(CH_2_)_3_OC_6_H_4_CH_2_-	SiMe_2_	20.8 (1.7)	−42		324	412	[56]

## Data Availability

Not applicable, as no new data were reported here.
